# A Mycological and Molecular Epidemiologic Study on Onychomycosis and Determination *In Vitro* Susceptibilities of Isolated Fungal Strains to Conventional and New Antifungals

**DOI:** 10.3389/fcimb.2021.693522

**Published:** 2021-07-15

**Authors:** Samaneh Halvaee, Roshanak Daie-Ghazvini, Seyed Jamal Hashemi, Sadegh Khodavaisy, Abbas Rahimi-Foroushani, Heidar Bakhshi, Zahra Rafat, Pegah Ardi, Mahdi Abastabar, Mahdi Zareei, Zeinab Borjian-Boroujeni, Hasti Kamali Sarvestani

**Affiliations:** ^1^ Department of Medical Parasitology and Mycology, School of Public Health, Tehran University of Medical Sciences, Tehran, Iran; ^2^ Food Microbiology Research Center, Tehran University of Medical Sciences, Tehran, Iran; ^3^ Department of Statistics and Epidemiology, School of Public Health, Tehran University of Medical Sciences, Tehran, Iran; ^4^ Department. of Medical Microbiology, School of Medicine, Guilan University of Medical Sciences, Rasht, Iran; ^5^ Invasive Fungi Research Center, Department of Medical Mycology and Parasitology, School of Medicine, Mazandaran University of Medical Sciences, Sari, Iran

**Keywords:** Onychomycosis, yeasts, dermatophytes, saprophytic agents, antifungal susceptibility testing, new antifungals, conventional antifungals

## Abstract

**Background:**

Onychomycosis is one of the most common and recurrent dermatological diseases worldwide. The antimycotic activity of prescribed medications varies according to the causative agents, and treatment failure rates exceeding 30%. This study aimed to assess the epidemiological profile of onychomycosis in Iran. Also, the susceptibilities to conventional and new antifungals were investigated.

**Methods:**

In this descriptive cross-sectional study, during the period of 18 months starting from September 2019 until March 2020, 594 nail specimens were obtained from patients who presented nail changes compatible with a clinical diagnosis of onychomycosis. The patients were referred from different cities, including Tehran, Kermanshah, Arak, Kashan, Rasht, Qom, Urmia, Zahedan, Hamadan, Zanjan, Borujerd, Bushehr, and Yazd. All the samples were subjected to microscopic examination and fungal culture. Fungi identified were confirmed through the PCR-sequencing method. The susceptibility to itraconazole, fluconazole, terbinafine, griseofulvin, posaconazole, ravuconazole, efinaconazole, luliconazole, and tavaborole was evaluated according to the Clinical and Laboratory Standards Institute (CLSI) guidelines, document M38-A2 for filamentous fungi, and document M27-A3 for yeasts.

**Results:**

594 patients were included. Of these, in 179 cases (30.1%) (95% CI:0.3 ± 0.037) onychomycosis was confirmed. The majority of patients were ≥ 60 years of age (n=58, 32.6%) and female (n=113, 63.1%). Saprophytic fungi accounted for the vast majority of the nail isolates (n=92, 51.4%) (95% CI:0.051 ± 0.0.073), followed by dermatophytes (n=45, 25.1%) (95% CI:0.25 ± 0.063), and yeasts (n=42, 23.5%) (95% CI:0.23 ± 0.061). Diabetes mellitus (77.3%), hypothyroidism (18.2%), and solid tumors (4.5%) were documented as the most prevalent underlying conditions. Antifungal susceptibility testing was performed against 60 fungal isolates (20 each of *Candida* species, saprophytic fungi, and dermatophytes). Efinaconazole, ravuconazole, and luliconazole were the most active agents against *Candida* species. Also, luliconazole, posaconazole, and efinaconazole were most potent against dermatophytes. Luliconazole had the greatest antifungal activity against saprophytic fungi.

**Conclusions:**

The prevalence of onychomycosis in Iranian patients was relatively high. LUL exhibited potent antifungal activity against the three groups of fungi tested, determining its broad-spectrum antimycotic activity and its probable use as the first-line therapy for onychomycosis.

## Introduction

Finger and toenails serve as visual advertisements of an individual’s overall health and have unquestionable effects on patients’ psychological, physical, social, and business activities. Onychomycosis defined as a fungal infection of the fingernails or toenails caused by yeasts, dermatophytes, and non-dermatophytic molds (Kaur et al., 2008; Rafat et al., 2019). Usually, it manifests as nail plate thickening (onychogryphosis), white or yellow nail discoloration, and separation of the nail from the nail bed (onycholysis). It is the most prevalent of all nail diseases and is considered an important public health problem affecting an increasing number of countries worldwide because of its prevalence and healthcare costs (Faergemann and Baran, 2003; Kaur et al., 2008; Westerberg and Voyack, 2013; Rafat et al., 2019). Its prevalence is estimated at more than 10% among healthy persons, and 40% in individuals older than 60 years, and accounts for 50% of all nail disorders seen in clinical practice (Baran et al., 1999). Several factors, including old age, underlying conditions (peripheral vascular disease, diabetes, compromised immune system, psoriasis, obesity, smoking), and walking shoeless in moist environments like public swimming pools and bathing places, are associated with an increased risk of this infection (Jacobsen and Tosti, 2017). There is also evidence suggesting that some people have a genetic predisposition to onychomycosis (Faergeman et al., 2005). Furthermore, different occupational and exercise activities (*i.e.*, ballet dance, gymnastics, water sports, tennis, soccer, and cricket), the incidence of nail injuries, the frequent use of synthetic clothing that retains moisture (sweat or precipitation), and communal bath/shower rooms are associated with a high risk for developing onychomycosis (Blake Steele et al., 2020). The accurate treatment of onychomycosis is essential as this infection has an important impact on the quality of life and could lead to a more serious infection and complication if left untreated (Scher and Baran, 2003). Due to the composition of the human nail plates, it acts as a formidable barrier against permeation and diffusion of all drugs. In addition, the nail has a slow growth rate, requiring a long duration of therapy, usually 8-12 months or longer, until the nail has grown back (Thomas et al., 2010). Conventional therapeutics (e.g., terbinafine, voriconazole, itraconazole, fluconazole, griseofulvin) have been the preferred treatment because of their accessibility and efficacy. Therapy failure is a substantial clinical problem occurring in 25–40% of patients with onychomycosis. This failure has been attributed to emerging resistant strains of dermatophytes, low bioavailability, the increasing prevalence of onychomycosis due to non-dermatophytes, the inability of topical antifungals to pass through the nail plate, and drug interactions (Buen et al., 2010). The new antifungals (posaconazole, ravuconazole, efinaconazole, luliconazole, tavaborole) serve as a further group of curative agents that might play important roles in the treatment of onychomycosis (Gupta et al., 2005). Lack of knowledge about antifungal susceptibility profiles of fungal elements causing onychomycosis against new antifungals among Iranian patients prompted us to conduct a comprehensive study to fill this gap. Therefore, the present study aimed to assess the epidemiological profile of onychomycosis in Iran and determine the susceptibilities to conventional and new antifungals. Clarifying this factor will aid in better clinical management and can help to select the best treatment protocols.

## Materials and Methods

### Ethics Statement

The study was approved by the Research Ethics Committee of Tehran University of Medical Sciences (the number of ethics committee protocol: IR.TUMS.SPH.REC.1399.114). All patients who agreed to participate in the investigation signed a written consent form.

### Patients, Sampling and Data Collection

This descriptive cross-sectional study was carried out over a period of 18 months from September 2019 to March 2020, in the Referral Medical Mycology Laboratory of Tehran University of Medical Sciences, Tehran, Iran. It is the main referral center of medical mycology in Iran, where especially patients with fungal infections who do not respond to routine treatments are referred for antifungal susceptibility testing to select the best treatment protocol. The patients were referred from different cities, including Tehran (n=61), Kermanshah (n=32), Arak (n=23), Kashan (n=18), Rasht (n=15), Qom (n=8), Urmia (n=5), Zahedan (n=4), Hamadan (n=3), Zanjan (n=3), Borujerd (n=3), Bushehr (n=2), and Yazd (n=2). Sampling was performed on patients who presented nail changes compatible with a clinical diagnosis of onychomycosis, including yellow or white discoloration of the nail, thickening or thinning of the nail, nail plate brittleness, and/or detached nail. The exclusion criteria were as follow: patients using topical and/or systemic antifungals at the time of sampling or up to 15 days before collecting the specimen, and patients whose clinical specimens were insufficient for complete analysis. Demographic features including age, gender, and underlying conditions were recorded. Scrapings were collected from the nails. The outermost debris collected was discarded, and fragments were taken from the site closest to the cuticle, where contains a greater concentration of fungal elements.

### Culture and Phenotypic Examination

The specimens were subjected to direct microscopy using 15% potassium hydroxide (KOH microscopy) and cultured on Sabouraud’s dextrose agar (SDA) containing 0.05 g/L chloramphenicol (Merck, Germany). The culture tubes were incubated at 30°C for up to 30 days. Fungal growth was assessed daily. Any growth obtained was identified by its identification characteristics include colony morphology, growth rate, and colony pigmentation.

Yeast isolates were identified based on formation and structural arrangement of chlamydospores on corn meal agar (Becton, France) and color of the colonies on CHROMagar™ *Candida* medium (CHROMagar, HiMedia, India). Potato-dextrose agar (PDA; Merck, Darmstadt, Germany) and Czapek agar (CZ, Micro media, Hungary) were used as differential media for the initial identification of isolated dermatophytes and non-dermatophytic filamentous fungi through colonial morphology and microscopic characteristics using lactophenol cotton blue and slide culture (Abe et al., 2009; Procop et al., 2017). For confirmation of identified species all isolates were subjected to PCR-sequencing as below.

### Molecular Technique

#### DNA Extraction

DNA was extracted using the High Pure PCR Template Preparation kit (Roche, Germany) according to the recommended instructions of the manufacturer.

#### PCR Conditions and Sequencing

To discriminate *Aspergillus* isolates at the species level the Beta tubulin gene of *Aspergillus* species was amplified using the forward (Bt2a: 5’-GGTAACCAAATCGGTGCTGCTTTC-3’) and reverse (Bt2b: 5-ACCCTCAGTGTAGTGACCCTTGGC-3) primers. Also, other fungal species were identified to the species level using the universal primers: ITS1 (5′TCC GTA GGT GAA CCT GCG G 3′), which hybridizes at the end of 18S rDNA, and ITS4 (5′TCC TCC GCT TAT TGA TAT GC 3), which hybridizes at the beginning of 28S rDNA (Life Technologies, Barcelona, Spain). The following thermal conditions were used: 95°C for 5 min, followed by 35 cycles of 30 s at 94°C, 60°C for 45 s, and 72°C for 1 min, followed by one final extension at 72°C for 5 min.

Positive PCR products were subjected to single-direction sequencing using a forward primer (Bioneer, South Korea). The amplicons were sequenced and results were analyzed using NCBI BLAST (https://www.blast.ncbi.nlm.nih.gov/Blast.cgi)n (http://its.mycologylab.org) database.

### Antifungal Susceptibility Testing (AFST)

*In vitro* antifungal susceptibility testing was performed against isolated strains according to the protocols described by the Clinical and Laboratory Standards Institute (CLSI) guidelines, document M38-A2 for filamentous fungi (Wayne and Clinical and Laboratory Standards Institute, 2008), and document M27-A3 for yeasts (Wayne, 2008). AFST included the following antifungal drugs: terbinafine (TER), voriconazole (VCZ), itraconazole (ITR), fluconazole (FLZ), griseofulvin (GSF), posaconazole (PSZ), ravuconazole (RAV), efinaconazole (EFIN), luliconazole (LUL), and tavaborole (TAVA) (all from Sigma-Aldrich, St. Louis, MO, U.S.A). The drug dilution ranges tested were 0.125–64 µg/mL for fluconazole and griseofulvin, 0.0625–32 µg/mL for tavaborole, 0.03125–16 µg/mL for voriconazole and itraconazole, 0.01562–8 µg/mL for terbinafine, and 0.003906-2 µg/mL for posaconazole, ravuconazole, efinaconazole, and luliconazole. Reference strains of *C. parapsilosis* (ATCC 22019) and *C. krusei* (ATCC 6258) were used for quality control purposes. Briefly, homogeneous conidial suspensions were spectrophotometrically measured at the 530 nm wavelength and a percent transmission within the range of 75-77%. The final inoculum suspension adjusted to 0.5-2.5 × 10^3^ conidia/mL in RPMI 1640 medium (GIBCO, UK) buffered at pH 7.0 with 0.165 M morpholino propanesulfonic acid (MOPS, Sigma-Aldrich, St. Louis, MO, USA). After adding 100 µl of the inoculum suspension the microdilution plates were incubated at 35°C for 48 h; the plates were read visually according to the recommendations proposed by the CLSI M27-A3 and M38-A2 documents.

### Statistical Analysis

We used SPSS software version 11 (SPSS Inc., Chicago, IL, USA) to analyze the data obtained in this study. The Relative percentages, 95% confidence interval, and Chi-square test were used to describe the antifungal susceptibility profile. We considered *P* values ≤ 0.05 to be statistically significant.

## Results

### Patient Characteristics

Between September 2019 and March 2020, 594 patients who passed the eligibility criteria were included in this study. Of this population, 179 (30.1%) (95% CI:0.3 ± 0.037) cases were diagnosed to suffer from onychomycosis. The 179 patients comprised 66 men (36.9%) and 113 women (63.1%) with a median age of 50 years (range 3 - 85 years).

Saprophytic fungi accounted for the vast majority of the nail isolates (n=92, 51.4%) (95% CI:0.051 ± 0.0.073), followed by dermatophytes (n=45, 25.1%) (95% CI:0.25 ± 0.063), and yeasts (n=42, 23.5%) (95% CI:0.23 ± 0.061). The highest prevalence of onychomycosis was found in the age group of ≥60 years (n=58, 32.6%) (**Table 1**). In the age group under 15 years only yeasts were the causative fungal agents of onychomycosis and in the age group above 60 years, dermatophytes were the predominant causative agent of onychomycosis (**Table 1**). The results showed the age of subjects was significantly effective on the prevalence of onychomycosis (*P* = 0.001).

**Table 1:** The frequency of causative agents of onychomycosis in regard to age groups.

Causative agent Age groups *(yrs)*	Yeasts		Saprophytic fungi		Dermatophytes		Total	
	number	%	number	%	Number	%	number	%
0-14	4	100	–	–	–	–	4	100
15-29	3	15.0	16	80.0	1	5.0	20	100
30-44	10	23.8	27	64.3	5	11.9	42	100
45-59	11	20.4	31	57.4	12	22.2	54	100
≥60	14	22.4	20	34.5	25	43.1	58	100
Total	42	23.5	92	51.4	45	25.1	179	100

**Figure 1:**
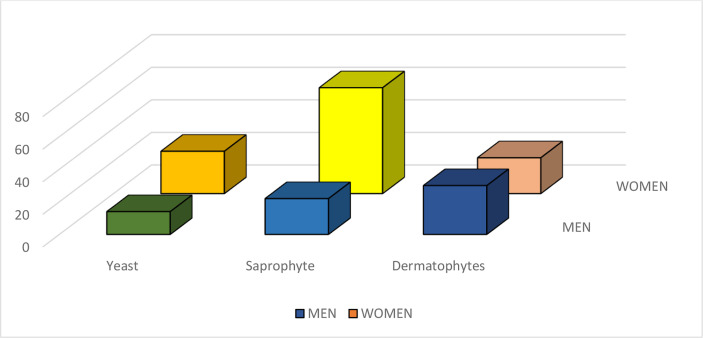
The frequency of causative agents of onychomycosis in regard to patient’s gender.

Results of the present study showed that there was no difference in the incidence of onychomycosis due to yeasts between females and males (*P* = 0.850958). On the other hand, significant gender differences in dermatophytic onychomycosis (*P* = 0. 000046) and onychomycosis due to saprophytic agents (*P* = 0.00022) were found between the male and female patients. Statistical analysis found a significant association between the causative agent of onychomycosis and the patient’s gender (*P< 0.00001*) (**Table 2** and **Figure 1**).

**Table 2:** The frequency of causative agents of onychomycosis in regard to patient’s gender.

Causative agent Gender	Yeasts		Saprophytic fungi		Dermatophytes		Total	
	number	%	number	%	number	%	Number	%
Male	16	24.3	22	33.3	28	42.4	66	36.9
Female	26	23.0	70	61.9	17	15.0	113	63.1
Total	42	23.5	92	51.4	45	25.1	179	100

*Trichphyton mentagrophytes* was the most frequently recovered dermatophyte (n=22, 48.9%), followed by *Trichphyton rubrum* (n=19, 42.2%), and *Trichphyton verrucosum* (n=4, 8.9%). Also, among yeast isolates responsible for onychomycosis, *Candida albicans* (n=22, 53.6%) was the most frequent detected species, followed by *Candida parapsilosis* (n=10, 24.4%), *Candidia tropicalis* (n=6, 13.9%), *Candida krusei* (n=2, 4.6%), and *Trichosporon asahii* (n=2, 4.6%). Furthermore, among saprophytic agents *Aspergillus flavus* (n=56, 60.9%) was the predominant etiologic agent of onychomycosis, followed by *Aspergillus niger* (n=13, 14.2%), *Aspergillus terreus* (n=8, 8.7%), *Fusarium oxysporum* (n=11, 11.9%), *Aspergillus candidus* (n=2, 2.2%), *and Nattrassia mangiferae* (n= 2, 2.2%). Phenotypic identification and sequencing results for the 179 fungal isolates included in the present study are shown in **Table 3**.

**Table 3:** Phenotypic and molecular identification data for 179 isolates included in this study.

Sample No.	Phenotypic Identification	Molecular identification (IT’S gene)	Molecular identification (Beta-tubulin gene)	GenBank accession number
1-20	*Candida albicans*	*Candida albicans*	**—**	MK793223, MT772046, MT772047, MT772052, MT772054, MT772056, MK138363, MT772070, KC905069, MT772073, MT772078, MT772079, MT772083, MT772085, MF614723, MT772090, MT772096, MG818819, MG818824, MG599201, MN4193373, MN419366 MW980770, MW980763
21,22	*Candida dubliniensis*			
23-32	*Candida parapsilosis*	*Candida parapsilosis*		MK394127, KY102205, KP131733, LN864530, MG241512, LC317527, DQ681358, EU564209, EU564205, KP131738
33-37	*Candida tropicalis*	*Candida tropicalis*		MK793225, MT772050, MK547223, MT772067, MT772080 MT772055
38	*Trichosporon sp.*			
39	*Candida glabrata*	*Candida krusei*		MH545928, FJ515204
40	*Candida krusei*			
41,42	*Trichosporon sp.*	*Trichosporon asahii*		AB018013, AB018014
43-56	Unidentifiable	*Trichophyton mentagrophytes*	—	HQ395066, HQ395067, HQ395068, HQ395069, HQ395070, HQ395071, AB520841, AB520842, AB520843, AB520844, AB520845, MK045530, MK045531, MK045532, MK045533, MK045534, MK045535, MK045536, MK045537, MK045538, MK045539, MK045540
57-60	*T. rubrum/T. mentagrophytes*			
61-64	*T. mentagrophytes*			
65-72	Unidentifiable	*Trichophyton rubrum*		FJ746657, FJ746658, FJ746659, FJ746660, GU291266, GU291267, GU291268, GU291296, GU291270, DW005385, DW00538, DW005357, DW005361, DW005363, DW005366, DW005368, DW005369, DW005373, AF291822
73-80	*T. rubrum*/*T*. *mentagrophytes*			
81-83	*T. rubrum*			
84	Unidentifiable	*Trichophyton verrucosum*		AF168126, AB 443930, AB 491473, KC 833516
85	*T. rubrum*/*T*. *mentagrophytes*			
86,87	*T. verrucosum*			
88-133	*Aspergillus flavus*	—	*Aspergillus flavus*	MK119732, MK119733, MK119734, MK119735, MK119736, MK119737, MK119738, MK119739, MK119740, MK119741, MK119742, MK119743, MK119744, MK119745, MK119746, MK119747, MK119748, MK119749, AY017536, AY017537, AY017538, AY017539, AY017540, AY017541, AY017542, AY017543, AY017544, AY017545, AY017546, AY017547, AY017548, AY017549, AY017550, AY017551, AY017552, AY017553, AY017554, AY017555, AY017556, AY017557, AY017558, AY017559, AY017560, AY017561, AY017562, AY017563, AY017564, M38265, M38257, M38258, M38265, M38260, M38261
134-143	*Aspergillus* sp.			
144-156	*Aspergillus niger*		*Aspergillusniger*	LC387867, LC387868, LC387869, LC387870, LC387871, LC387872, LC387873, LC387874, LC387875, LC387876, LC387877, LC387878, LC387879
157-161	*Aspergillus terreus*		*Aspergillus terreus*	GQ461911, GQ461912, GQ461913, GQ461914, GQ461915, GQ461916, GQ461917, GQ461918
162-164	Unidentifiable			
165-174	*Fusarium* sp.	*Fusarium oxysporum*	—	GQ922558, GQ922559, GQ922560, GQ922561, GQ922562, GQ922563, GQ922564, GQ922565, GQ922566, GQ922567, GQ922568,
175	Unidentifiable			
176,177	*Aspergillus* sp.	*Aspergillus candidus*		FN907924, FN907925
178,179	*Nattrassia* sp.	*Nattrassia mangiferae*		MZ377100, MT010216

Diabetes mellitus, hypothyroidism, and solid tumors were found in 77.3%, 18.2%, and 4.5% of patients, respectively. These predisposing factors were not significantly associated with the prevalence of onychomycosis (*P* = 0.087).

### Antifungal Susceptibility Testing

#### Antifungal Activity Against Candida Species

**Figure 2:**
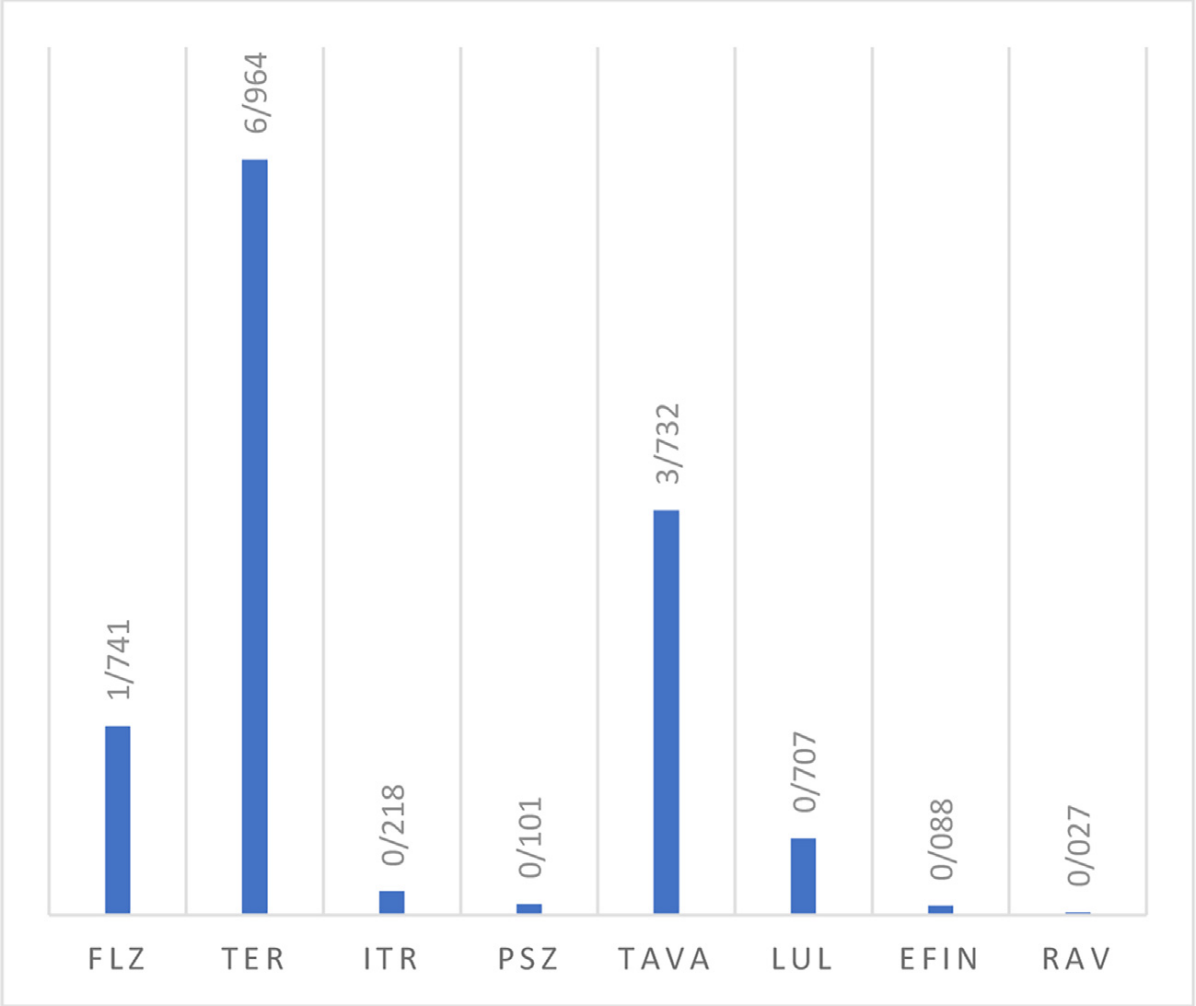
The geometric mean obtained by testing the susceptibility of yeast isolates to each antifungal agent.

*In vitro* activity based on MIC ranges, geometric mean (GM), 50% MIC (MIC50), and 90% MIC (MIC90) of all tested antifungals against the isolated yeast species responsible for onychomycosis is presented in **Table 4**. When all *Candida* strains were considered together, it was found that they were highly susceptible to EFIN (MIC range: 0. 0078-0.5 µg/mL), LUL (MIC range: 0.0125-2µg/mL), and RAV (MIC range: 0.0039-0.25/25 µg/mL) whereas TER (MIC range: 4->8 µg/mL), TAVA (MIC range: 2-8 µg/mL), and FLZ (MIC range: 0.25-16 µg/mL) had the lowest antifungal activity against *Candida* species (**Figure 2**).

**Table 4:** The geometric mean, MIC ranges, MIC50 and MIC90 values obtained by testing the susceptibility of yeast isolates to each antifungal agent.

Strains	Antifungals	MIC range(µg/mL)	MIC 50 (µg/mL)	MIC 90(µg/mL)	GM(µg/mL)	Mean	V	SD	CI 95%
*Candida* species (n=20)	FLZ	0.25-16	1	16	1.741	5.325	48.90	6.993	2.05218, 8.59782
	TER	4->8	8	8	6.964	7.200	2.560	1.600	6.451, 7.949
	ITR	0.0625-2	0.125	1	0.218	0.412	0.312	0.558	0.15085, 0.67315
	PSZ	0.0078-2	0.0625	1	0.101	0.343	0.336	0.580	0.07155, 0.61445
	TAVA	2-8	4	8	3.732	4.00	2.40	1.550	3.27, 4.73
	LUL	0.0125-2	0.5	2	0.707	1.062	0.635	0.797	0.68899, 1.43501
	EFIN	0. 0078-0.5	0.0625	0.5	0.088	0.202	0.042	0.206	0.10559, 0.29841
	RAV	0.0039-0.25	0.0156	0.125	0.027	0.059	0.006	0.076	0.02343, 0.09457

GM, Geometric mean; MIC, Minimum inhibitory concentration; MIC50, minimal concentration that inhibits 50% of isolates; MIC90, minimal concentration that inhibits 90% of isolates; TER, terbinafine; ITR, itraconazole; FLZ, fluconazole; PSZ, posaconazole; RAV, ravuconazole; EFIN, efinaconazole; LUL, luliconazole; TAVA, tavaborole; V, Variance; SD, standard deviation; and CI, confidence interval.

#### Antifungal Activity Against Dermatophyte Species

**Figure 3:**
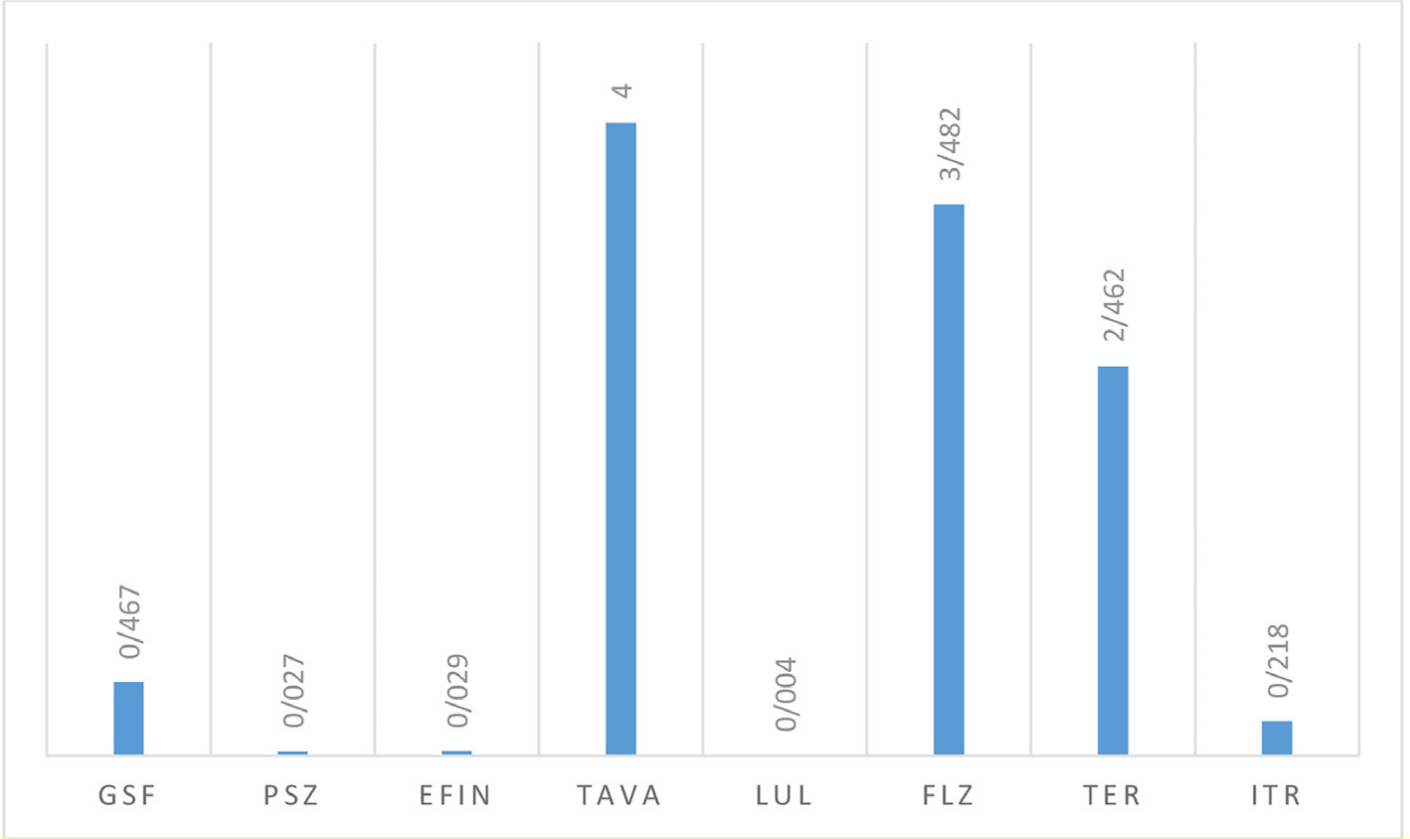
The geometric mean obtained by testing the susceptibility of dermatophytic isolates to each antifungal agent.

**Table 5** lists the GM, MIC50, and MIC90 of each antifungal agent against the isolated dermatophytes responsible for onychomycosis in the present study. Totally, dermatophyte species were highly susceptible to LUL (MIC range: <0.0039 µg/mL), PSZ (MIC range: 0. 0078-0.0625 µg/mL), EFIN (MIC range: 0. 0625-0.0156 µg/mL), ITR (MIC range: 0. 125-0.5 µg/mL) and GSF (MIC range: 0.25-1µg/mL) whereas TER (MIC range: 1-4 µg/mL), TAVA (MIC range: 2-8 µg/mL), and FLZ (MIC range: 1-32 µg/mL) had the lowest antifungal activity against these fungal species (**Figure 3**).

**Table 5:** The geometric mean, MIC ranges, MIC50 and MIC90 values obtained by testing the susceptibility of dermatophytes to each antifungal agent.

Strains	Antifungals	MIC range (µg/mL)	MIC 50 (µg/mL)	MIC 90 (µg/mL)	GM (µg/mL)	Mean	V	SD	CI95%
Dermatophytes (n=20)	ITR	0. 125-0.5	0.125	0.5	0.218	0.275	0.034	0.184	0.18889, 0.36111
	TER	1-4	2	4	2.462	2.8	1.56	1.249	2.215, 3.385
	FLZ	1-32	2	16	3.482	7	88.8	9.423	2.59, 11.41
	LUL	<0.0039	0.0039	0.0039	0.004	0.0039	0	0	0.0039, 0.0039
	TAVA	2-8	4	8	4.000	4.6	5.64	2.375	3.488, 5.712
	EFIN	0. 0625-0.0156	0.0312	0.0625	0.029	0.033	0.0003	0.016	0.02551, 0.04049
	PSZ	0. 0078-0.0625	0.0312	0.0625	0.027	0.034	0.0004	0.020	0.02464, 0.04336
	GSF	0.25-1	0.5	1	0.467	0.525	0.068	0.261	0.40285, 0.64715

GM, Geometric mean; MIC, Minimum inhibitory concentration; MIC50, minimal concentration that inhibits 50% of isolates; MIC90, minimal concentration that inhibits 90% of isolates; TER, terbinafine; ITR, itraconazole; FLZ, fluconazole; GSF, griseofulvin; PSZ, posaconazole; EFIN, efinaconazole; LUL, luliconazole; TAVA, tavaborole; V, Variance; SD, standard deviation; and CI, confidence interval.

#### Antifungal Activity Against Saprophytic Fungi

**Figure 4:**
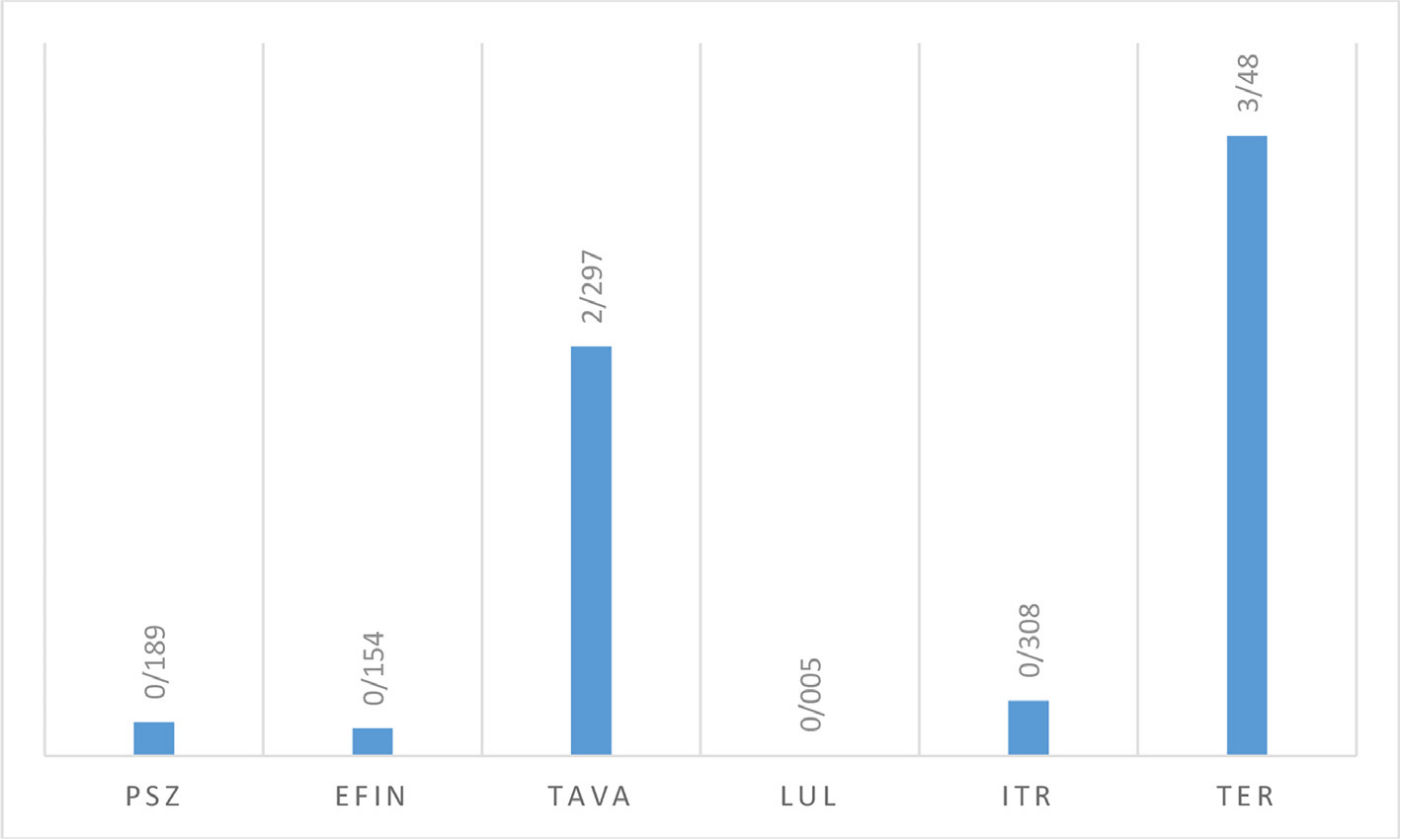
The geometric mean obtained by testing the susceptibility of saprophytic isolates to each antifungal agent.

*In vitro* activity based on MIC ranges, geometric mean (GM), 50% MIC (MIC50), and 90% MIC (MIC90) of all antifungals against saprophytic fungi responsible for onychomycosis is shown in **Table 6**. The results showed that LUL (MIC range: <0.0039 µg/mL) had the greatest and TER (MIC range: 1->8 µg/mL), and TAVA (MIC range: 2-8 µg/mL) had the lowest antifungal activity against saprophytic fungi (**Figure 4**).

**Table 6:** The geometric mean, MIC ranges, MIC50 and MIC90 values obtained by testing the susceptibility of saprophytic fungi to each antifungal agent.

Strains	Antifungals	MIC range(µg/mL)	MIC 50 (µg/mL)	MIC 90 (µg/mL)	GM (µg/mL)	Mean	V	SD	CI95%
Saprophytic fungi (n=20)	TER	1->8	4	4	3.48	3.9	2.89	1.7	3.104, 4.696
	ITR	0.0625-16	0.25	0.5	0.308	1.806	22.397	4.733	0.40911, 4.02111
	LUL	<0.0039	0.0039	0.0039	0.005	0.01	0.0003	0.018	0.0016, 0.0184
	TAVA	2-8	2	2	2.297	2.6	3.24	1.8	1.758, 3.442
	EFIN	0.0078-1	0.25	0.25	0.154	0.257	0.069	0.262	0.13438, 0.37962
	PSZ	0.156-1	0.25	1	0.189	0.352	0.123	0.350	0.18819, 0.51581

GM, Geometric mean; MIC, Minimum inhibitory concentration; MIC50, minimal concentration that inhibits 50% of isolates; MIC90, minimal concentration that inhibits 90% of isolates; TER, terbinafine; ITR, itraconazole; PSZ, posaconazole; EFIN, efinaconazole; LUL, luliconazole; TAVA, tavaborole; V, Variance; SD, standard deviation; and CI, confidence interval.

## Discussion

Onychomycosis is an important global disease and represents 20% to 40% of all onychopathies. Although it has worldwide occurrence, its frequency is variable which depends on different climatic, ethics, professional, and socio-economic conditions. In the present study, from 594 patients suspected to onychomycosis, 179 cases (30.1%) were positive based on laboratory finding, while a comprehensive survey conducted by Pönnighaus in Africa did not find any cases of onychomycosis (Pönnighaus et al., 1996), also in a study conducted by Roberts et al., in the United Kingdom the estimated prevalence of onychomycosis was 1.3 to 4.7% (Roberts, 1992). The present study assessed that onychomycosis is extremely rare in children, common in adults, and very frequent in the elderly. The finding is according to other studies done in the world (Smith et al., 2001; Htwe et al., 2007; Abdullah and Abbas, 2011). This increased prevalence of onychomycosis in the elderly (≥ 60 years of age) may be associated with the presence of arthritis and other related conditions that weaken physical activity and flexibility. It should be noted that this age group is more susceptible to colonization by infectious organisms resulting from inadequate foot and nail hygiene.

The results of the present study showed that females were affected more than males by onychomycosis. One reason is that for any nail disorder, females go to the doctor more often than males. Also, prolonged contact with water and soap or other detergents in females is another risk factor that decreases the local immunity at the nail complex (Jayatilake et al., 2009).

Our results on the distribution of onychomycosis etiological agents differ from other published reports. In the present study, saprophytic fungi accounted for the vast majority of the nail isolates, while an Iranian meta-analysis study speculated that yeasts are the predominant leading agent of onychomycosis (Rafat et al., 2019). Also, a multicenter study performed in the USA reported that the commonest causative agents of onychomycosis are dermatophytes (Ghannoum et al., 2000). As our results show, among yeasts, *Candida albicans* was the most prevalent species isolated from patients with onychomycosis, and among dermatophytes, *Trichphyton mentagrophytes* was the main species isolated from the patients. Furthermore, the predominant isolated saprophytic fungi in the present study was *Aspergillus flavus*. These findings are in accordance with the results of a systematic review and meta-analysis conducted by Rafat et al., in Iran (Rafat et al., 2019). Furthermore, diabetes mellitus and hypothyroidism were observed as the risk factors for developing onychomycosis in the present study. Previous studies indicated that endocrine disorders (hypothyroidism and diabetes mellitus) are some of the common systemic diseases that facilitate fungal nail infections *via* suppression of host immunity (Chi et al., 2005; Jayatilake et al., 2009).

Itraconazole, terbinafine, and fluconazole are the most widely available antifungal agents used for the systemic treatment of onychomycosis. Historically, the treatment of onychomycosis has been challenging (Gupta and Versteeg, 2016). In the current study, we used a panel of different species of dermatophytes, yeasts, and saprophytic fungi isolated from patients with onychomycosis to evaluate the *in vitro* activity of new antifungals (posaconazole, ravuconazole, efinaconazole, luliconazole, tavaborole) in comparison with conventional therapeutics (terbinafine, itraconazole, fluconazole, and griseofulvin). The high MICs obtained for *Candida* species with terbinafine, tavaborole, and fluconazole are consistent with those previously reported (Darkes et al., 2003; Sojakova et al., 2004; Buen et al., 2010; Abastabar et al., 2018) therefore, we would recommend cautious use of these antifungals against different *Candida* strains causing onychomycosis. Also, in the present study, 10% of *Candida* isolates were resistant to posaconazole and itraconazole. In contrast, the results of a study conducted by Sabatelli et al., showed that posaconazole was frequently more active than itraconazole, fluconazole, voriconazole, and amphotericin B against approximately 7,000 isolates of *Candida* and *Cryptococcus* spp (Sabatelli et al., 2006). Furthermore, in the present study, LUL, EFIN, and RAV had low MICs against *Candida* species and it was found that they were highly susceptible to these antifungals. This finding is consistent with the results from previous reports (Pfaller et al., 2002; Uchida et al., 2004; Siu et al., 2013).

Relative to the other agents tested, LUL possessed the highest antifungal activity against all dermatophytes. This finding is in agreement with previous studies (Uchida et al., 2004; Baghi et al., 2016). Also, the results of the present study showed that dermatophytes were highly susceptible to PSZ, EFIN, ITR, and GSF, whereas TER, TAVA, and FLZ had the lowest antifungal activity against these fungal species. In accordance, in previous studies, against dermatophytes, low MICs were obtained with EFIN, and PSZ (Gupta et al., 2005; Siu et al., 2013). Results of a study conducted by Baghi et al., demonstrated TER, and ITR displayed excellent activity against all dermatophyte isolates, although they reported that the majority of dermatophyte isolates showed low susceptibility to GSF and very low to FLZ (Baghi et al., 2016).

Our results showed that LUL had the greatest and, TER and TAVA had the lowest antifungal activity against saprophytic fungal strains isolated from onychomycosis. In accordance, another study has been reported that LUL had strong *in vitro* antifungal activity against *Aspergillus fumigatus* (Ghannoum et al., 2000). In the present study, MICs of TAVA against saprophytic fungal strains were similar to those reported by Abastabar et al. (2018), and MICs of TER against these fungal isolates were similar to those reported by Mao et al. (2006). Also, the resistance of saprophytic fungi to ITR and PSZ was accounted for 10% and 20%, respectively. In a study conducted by Gupta et al., the lowest MICs for saprophytic fungal strains was obtained with posaconazole, followed by, ravuconazole, terbinafine, and itraconazole (Jayatilake et al., 2009).

## Conclusion

In conclusion, our results provide further evidence for the spectrum and potency of luliconazole, a novel topical imidazole, in the treatment of onychomycosis. In the present study, luliconazole had low MICs against the three groups of fungi tested (yeasts, dermatophytes, and non-dermatophytic molds) determining its broad-spectrum antimycotic activity and its probable use as the first-line therapy for onychomycosis.

## Data Availability Statement

The raw data supporting the conclusions of this article will be made available by the authors, without undue reservation.

## Ethics Statement

The studies involving human participants were reviewed and approved by Tehran University of Medical Sciences. Written informed consent to participate in this study was provided by the participants’ legal guardian/next of kin.

## Author Contributions

SH, PA, ZB, MZ, MA, and HKS performed experiments. RD, SJH, and SK supervised the study. HB and AR performed the statistical analysis. ZR wrote the manuscript. SH, PA, ZB, MZ, MA, and HKS performed experiments. RD obtained study funding. All authors contributed to the article and approved the submitted version.

## Funding

This work was supported by the funding from Tehran University of Medical Sciences, Tehran, Iran.

## Conflict of Interest

The authors declare that the research was conducted in the absence of any commercial or financial relationships that could be construed as a potential conflict of interest.
